# Frequency-specific cortical swallowing-network responses following neuroengineering-guided individualized multichannel tDCS

**DOI:** 10.1186/s12984-026-02214-0

**Published:** 2026-11-12

**Authors:** Paul Muhle, Asad Khan, Anne Jung, Nikolas Vogenauer, Andreas Wollbrink, Joachim Gross, Bendix Labeit, Rainer Dziewas, Carsten H. Wolters, Sonja Suntrup-Krueger

**Affiliations:** 1 Department of Neurology, University Hospital Muenster, Albert-Schweitzer-Campus 1 A, 48149 Muenster, Germany; 2 Institute for Biomagnetism and Biosignalanalysis, University Hospital Muenster, Malmedyweg 15, 48149 Muenster, Germany; 3 Department of Psychology and Neurosciences, Leibniz-Institut für Arbeitsforschung an der TU Dortmund, Dortmund, Germany; 4 Otto Creutzfeldt Center for Cognitive and Behavioral Neuroscience, University of Münster, Münster, Germany; 5 Department of Neurology, Medical Faculty, Heinrich Heine University Duesseldorf, Duesseldorf, Germany; 6 Department of Neurology, Klinikum Osnabrück, Am Finkenhügel 1, 49076 Osnabrück, Germany

**Keywords:** Dysphagia, Precision neuromodulation, Multichannel transcranial direct current stimulation, Neuroengineering, Magnetoencephalography, Rehabilitation

## Abstract

**Background:**

Neuromodulatory interventions for dysphagia are increasingly explored as adjunctive rehabilitation strategies, yet their efficacy may be limited by non-individualized stimulation protocols and insufficient consideration of network-specific brain dynamics. Advances in neuroengineering now enable individualized targeting based on functional neuroimaging and computational modeling, offering the potential for precision neuromodulation of swallowing-related brain networks.

**Methods:**

We investigated whether individualized multichannel transcranial direct current stimulation (mc-tDCS), optimized using magnetoencephalography (MEG)-derived target localization, MRI-based finite-element head modeling, and a distributed constrained maximum intensity (D-CMI) optimization framework, modulates the cortical swallowing network. In a randomized sham-controlled crossover design, eight healthy adults underwent three stimulation conditions: low-intensity mc-tDCS (1.5 mA), high-intensity mc-tDCS (4.0 mA), and sham stimulation. Swallowing-related cortical activity was assessed before and after stimulation using MEG source reconstruction of event-related desynchronization during uncued voluntary swallowing and a visually cued swallowing task requiring precise timing. A repeated-measures Dose × Task × Time model tested the hypothesized interaction separately in five frequency bands, with Holm correction across bands.

**Results:**

No Dose × Task × Time interaction was significant after Holm correction across the five frequency bands (all adjusted *p* ≥ 0.274). Within-condition pre–post analyses identified alpha- and beta-band changes after 1.5-mA stimulation during challenged swallowing and a low-gamma change after 4.0-mA stimulation during uncued swallowing. Challenged-task timing accuracy increased over time, without significant Dose or Dose × Time effects.

**Conclusions:**

This study demonstrates the feasibility of integrating individualized MEG-based targeting, subject-specific head modeling, and optimized multichannel stimulation. The hypothesized Dose × Task × Time interaction was not significant. Exploratory within-condition pre–post differences were observed in alpha, beta, and low-gamma activity, but do not establish stimulation-specific effects. Larger controlled studies are needed to evaluate the physiological effects of this individualized approach.

**Supplementary Information:**

The online version contains supplementary material available at https://doi.org/10.1186/s12984-026-02214-0.

## Introduction

Swallowing is a complex sensorimotor function governed by a distributed network comprising cortical, subcortical, and brainstem structures [1, 2]. Disruption of this network results in dysphagia, a frequent and clinically significant complication of neurological disease, particularly after stroke, and a major contributor to morbidity, mortality, and healthcare costs due to complications such as aspiration pneumonia and malnutrition [3, 4]. Despite its high prevalence and clinical impact, effective therapeutic options for dysphagia remain limited, highlighting the need for novel, mechanism-based treatment strategies. In this context, non-invasive brain stimulation has emerged as a promising approach to modulate dysfunctional brain networks and support neurorehabilitation [3, 5, 6].

Transcranial direct current stimulation (tDCS) is a widely used non-invasive neuromodulation technique that can facilitate synaptic plasticity and motor learning processes [7–9]. In the domain of swallowing, early studies demonstrated that anodal tDCS increases excitability of cortical swallowing representations [9]. Subsequent work showed that tDCS induces bilateral, frequency-specific modulation of swallowing-related cortical activity, accompanied by behavioral improvements during a swallowing task requiring precise timing in response to visual cues [10]. These neurophysiological effects translate into clinically relevant outcomes, as demonstrated in randomized controlled trials in dysphagic stroke patients, where repeated tDCS enhanced functional recovery compared to sham stimulation [11]. Meta-analyses support an overall beneficial effect of tDCS on swallowing function but consistently report substantial heterogeneity in stimulation protocols and effect sizes, underscoring the need for more individualized and mechanistically informed approaches [3, 12].

A key limitation of conventional tDCS in this context is its reliance on standard bipolar montages, which may be suboptimal for modulating distributed sensorimotor networks. Bipolar stimulation produces relatively diffuse electric fields, potentially leading to co-stimulation of non-target regions and reduced focality at the intended cortical site [13]. Moreover, conventional approaches typically neglect interindividual variability in anatomy, functional organization, and tissue conductivities, all of which critically shape the spatial distribution and orientation of induced electric fields [13–15]. These limitations are particularly relevant for complex functions such as swallowing, where cortical representations are distributed, dynamically organized, and subject to substantial inter- and intra-individual variability. More broadly, a central unresolved challenge in neurotherapeutics is how to define stimulation “dose” at the level of brain networks rather than in terms of applied current intensity alone. Emerging evidence suggests that neuromodulatory effects depend on interactions among electric field properties, target orientation, and the ongoing functional state of the network [5–8], 13, 16–21]. However, current clinical protocols rarely systematically incorporate these factors, which may contribute to treatment effect variability and limited reproducibility.

To address these limitations, multichannel tDCS (mc-tDCS) approaches have been developed that enable individualized, model-based optimization of current distribution. Distributed constrained maximum intensity (D-CMI) optimization allows the generation of subject-specific electrode configurations that maximize electric field intensity along a predefined target orientation while distributing currents across multiple electrodes to improve tolerability and focality [22]. Proof-of-principle work has demonstrated that this approach can outperform conventional bipolar stimulation in modulating cortical targets, resulting in enhanced source-level effects [23]. Building on these advances, the present proof-of-concept study investigated whether a neuroengineering-guided approach integrating MEG-based target localization, subject-specific finite-element head modeling, and D-CMI optimized multichannel stimulation can modulate the cortical swallowing network. We hypothesized that individualized mc-tDCS would induce measurable frequency-specific changes in swallowing-related cortical activity and tested whether these changes showed a Dose × Task × Time interaction. Because the two tasks differed in several features, we interpreted any task-specific pattern at the experimental task-condition level rather than as a single isolated physiological state.

## Methods

### Experimental outline and participants

We investigated the effects of individually optimized multichannel tDCS (mc-tDCS) at different stimulation intensities on cortical swallowing activity in healthy adults in a randomized, sham-controlled crossover design. Eight participants (mean age: 29.6 ± 7.9 years; 4 females) were included. None had a history of neurological, psychiatric, or otolaryngological disorders or were taking medication affecting the central nervous system. The local ethics committee approved the study protocol, and all participants provided written informed consent.

**Fig. 1:**
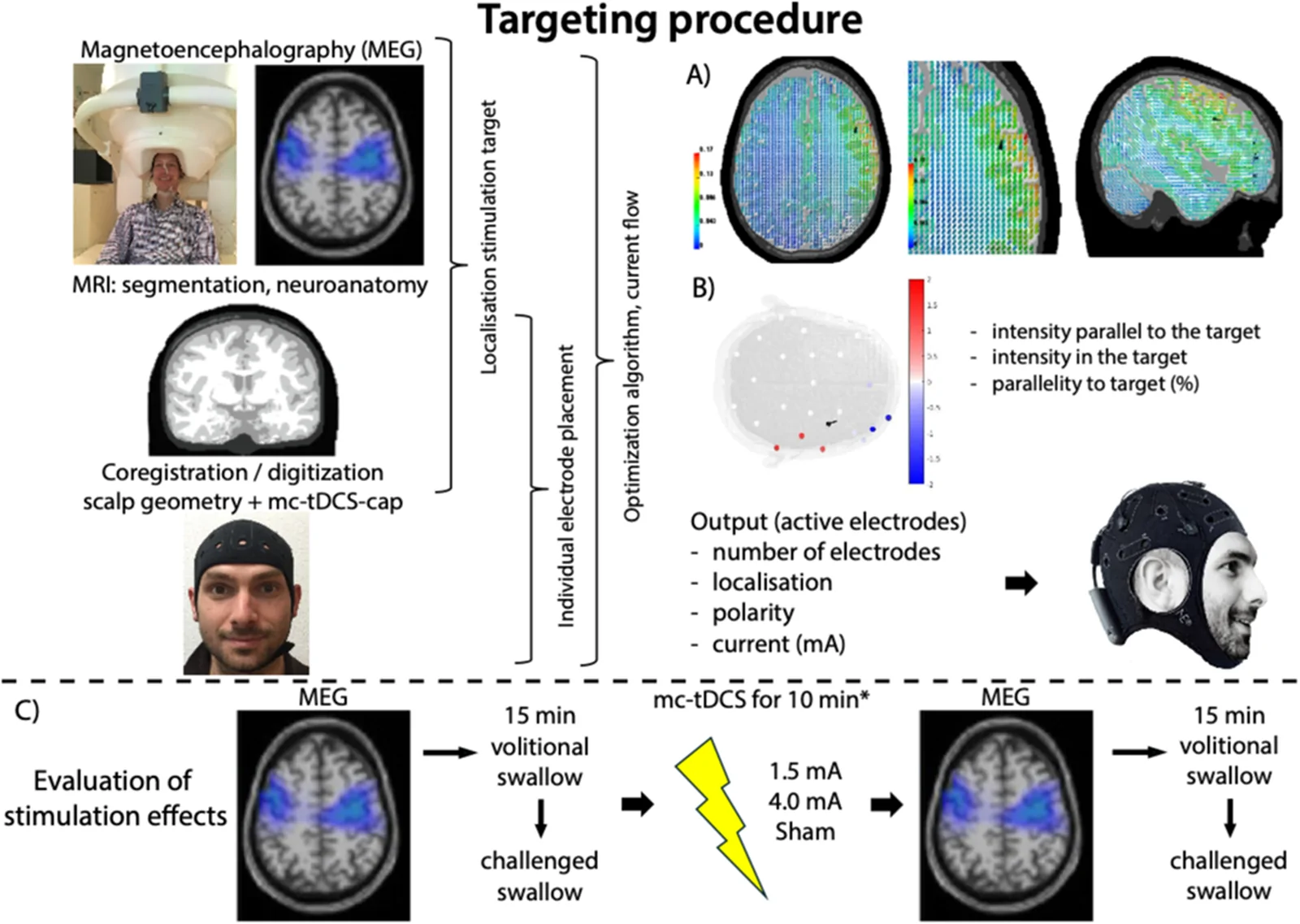
Experimental workflow for individualized multichannel tDCS targeting and network-level assessment (**A**) Swallowing-related cortical activity was recorded using magnetoencephalography (MEG) during voluntary uncued swallowing. Source reconstruction was used to identify the individual cortical swallowing target. (**B**) Individualized multichannel tDCS (mc-tDCS) montages were optimized based on subject-specific anatomical MRI and finite element modeling using a distributed constrained maximum intensity (D-CMI) approach. (**C**) Participants underwent three randomized stimulation sessions (low-intensity mc-tDCS, high-intensity mc-tDCS, sham). Swallowing-related cortical activity was assessed before and after stimulation using MEG during uncued and challenged swallowing tasks

The study was divided into two parts: targeting and stimulation (see Fig. 1 for workflow). First, we recorded a 15-minute uncued voluntary swallowing MEG measurement in each participant. Using MEG source analysis, we reconstructed an individual right-hemispheric cortical stimulation target within the broader swallowing network. The temporal analysis window encompassed the swallowing event around peak submental EMG activity and did not permit phase-specific attribution. After reconstructing this target individually for each subject, we optimized a personalized mc-tDCS montage using each subject’s segmented anatomical MRI, digitized head shape, and a coregistered electrode cap, along with the previously established D-CMI algorithm.

In the stimulation phase, participants completed three randomized, sham-controlled crossover sessions: individually optimized anodal mc-tDCS at 1.5 mA, anodal mc-tDCS at 4 mA, and sham stimulation, with observed inter-session intervals of 8–77 days (median 35 days). Swallowing-associated cortical network activity was recorded with MEG before and after mc-tDCS during uncued voluntary swallowing and a visually cued challenged task requiring swallowing within a 150-ms target interval [10]. The experimental factor Task therefore contrasted two task conditions that differed simultaneously in external cueing, temporal precision, performance monitoring, immediate feedback, and attentional demand; it was not intended to isolate any one of these dimensions. The after-effects for each tDCS condition were statistically analyzed and compared. The study was designed as a mechanistic proof-of-concept investigation.

### Magnetoencephalography (MEG)

#### MEG data acquisition

We used a 275-channel MEG system (Omega 275, CTF Systems Inc.) to identify cortical activity during swallowing. Participants were positioned upright, facing a screen approximately 80 cm in front of them, and instructed to keep their eyes open during the MEG session. To facilitate swallowing, still water was continuously infused at a slow rate of 8–12 ml/minute into the mouth via a plastic tube. Magnetic-field data were acquired at 600 Hz and online low-pass filtered at 150 Hz.

Submental sEMG was recorded synchronously at 600 Hz using a bipolar pair of Ag/AgCl skin electrodes, with one electrode over the left and one over the right submental muscle group, and a separate ground electrode over the clavicle. Signals were recorded using a DSQ 2017E bipolar EOG/EMG amplifier (CTF Systems Inc., Canada; nominal gain 1) and high-pass filtered at 0.1 Hz. For the exploratory peripheral endpoints, manually identified swallow onset and offset defined the interval used to calculate swallow duration, peak-to-peak amplitude, and RMS amplitude. Amplitudes were baseline-corrected using the 1-s interval preceding swallow onset and converted to microvolts. Events that could not be reliably delineated because of substantial artifacts were excluded during visual review. No additional filtering, automated artifact rejection, rectification, smoothing, or normalization was applied. Because these non-normalized amplitudes depend on electrode placement and individual tissue properties, they were interpreted only as exploratory within-subject measures. Stimulus and sEMG events were synchronized with the MEG acquisition [10, 24–27].

For MEG target reconstruction, we recorded voluntary uncued swallowing for 15 min. To assess intervention effects, the uncued task was repeated and followed by a previously developed challenged swallowing task requiring precisely timed swallow initiation in response to visual cues (10 min; approximately 40 trials) [10, 26]. The task order was fixed across visits and was not counterbalanced. After stimulation, uncued swallowing therefore occupied approximately 5–20 min and challenged swallowing approximately 20–30 min after the end of mc-tDCS. A trial, including one with multiple detected swallow onsets, was classified as on time if the first identifiable onset occurred within the 150-ms target interval. Trials without an identifiable swallow onset were treated as missing. Participants received immediate feedback indicating “too early,” “on time,” or “too late.” Visual stimuli and feedback were delivered using Presentation software (NeuroBehavioral Systems, Inc., USA).

#### MEG preprocessing and source reconstruction

MEG data were analyzed using an established pipeline designed for our swallowing tasks and implemented in custom MATLAB scripts within the FieldTrip toolbox (http://www.ru.nl/fcdonders/fieldtrip) [10, 24–29]. This process included frequency filtering across the theta (4–8 Hz), alpha (8–13 Hz), beta (13–30 Hz), low gamma (30–60 Hz), and high gamma (60–80 Hz) bands. For individualized targeting, the 15-minute uncued swallowing recording was reconstructed separately with an LCMV beamformer on a 3-mm grid. The resulting functional source estimate, together with the participant’s individual anatomical MRI, was transferred to the modeling team for FEM/D-CMI montage optimization. This targeting-specific 3-mm reconstruction was independent of the regular pre/post MEG analyses reported here, which were computed on a 10-mm source grid. For the regular analyses, an LCMV beamformer was applied to estimate swallow-related event-related desynchronization (ERD) [10]. ERD was calculated in a time window of − 0.4 to + 0.6 s relative to peak sEMG swallowing activity, using a 1-s post-swallow baseline. The targeting reconstruction informed individualized montage optimization; separately, the 10-mm volumetric source maps were used for the group-level statistical analyses. The primary outcome measure was the change in source-level swallowing-related ERD within the predefined cortical swallowing network. Modulation effects were examined across stimulation intensities and task states. Frequency-band–specific and spatially resolved effects were treated as exploratory [10, 24–30].

#### Statistical analysis of MEG data

For pre–post comparisons of intervention effects, each subject’s volumetric source estimates were spatially normalized to a template MNI brain (T1, Montreal Neurological Institute, Canada) using SPM8 (http://www.fil.ion.ucl.ac.uk/spm). Grand averages of normalized and realigned source activation maps were calculated for all time points, conditions, and frequency bands. The region of interest (ROI) comprised cortical regions known to be involved in swallowing (Supplementary Table S1) [10, 25, 31]. Cluster-based nonparametric randomization implemented in FieldTrip was used to identify source locations that were modulated in separate within-condition pre–post comparisons (*p* < 0.05), controlling for multiple spatial comparisons within the predefined ROI [32]. To test the hypothesized interaction directly, ROI-mean ERD values were entered into a repeated-measures model with factors Dose (1.5 mA, 4.0 mA, sham), Task (challenged, uncued), and Time (pre, post), separately for theta, alpha, beta, low-gamma, and high-gamma bands. The primary term was Dose × Task × Time; Greenhouse–Geisser-adjusted p values were used when available and Holm correction was applied across the five bands. The separate cluster-based pre–post findings were treated as exploratory descriptive results and not as formal evidence of state- or intensity-dependence. As an exploratory crossover sensitivity check, the ROI-mean pre–post change in the three reported frequency-band/task combinations was modeled with participant, current dose, and session period. The joint Period tests were Holm-adjusted across these three checks.

### MRI acquisition

Structural MRI data acquired from a MAGNETOM Prisma 3.0 T Scanner (Siemens Medical Solutions, Erlangen, Germany) provided realistic head models for the mc-tDCS optimization procedure [23, 33]. In brief, gadolinium markers were placed at the nasion, left, and right external auditory canal openings (ear canal fiducials) for landmark-based co-registration purposes. Imaging included T1-weighted Imaging: High-resolution (1 mm³) anatomical scans using a fast gradient-echo sequence for scalp, gray, and white matter segmentation; T2-weighted Imaging: Segmentation of the compact and spongy layers of the skull. Diffusion-weighted Imaging: Acquiring for white matter anisotropy modeling with diffusion sensitivity values (b = 0 and 1000 s/mm²) in 20 directions, uniformly distributed on a sphere.

### Targeting and generation of mc-tDCS montages

#### Individualized head modeling and montage optimization

To compute individually optimized mc-tDCS montages for stimulation of the cortical swallowing target, dipole location and orientation at the individual right-hemispheric activation peak within the swallowing network were taken from the MEG source estimates. Skull-conductivity-calibrated realistic head models were generated from each participant’s MRI, as previously published [23, 33]. In brief, a compartment head model for each subject, representing the tissues scalp, skull compacta, skull spongiosa, cerebrospinal fluid, gray matter, and white matter, was generated. Geometry-adapted hexahedral finite element method (FEM) modeling [34] of the segmented head model was carried out using SimBio-VGRID.1 (a generic environment for bio-numerical simulations www.simbio.de), and different tissue conductivities were modelled.

The 39 possible electrode positions of the mc-tDCS cap (Starstim-8 cap, Neuroelectrics, Barcelona, Spain) corresponding to the international 10/20 EEG system were digitized with each individual’s headshape and anatomical landmarks (nasion, left, and right external auditory canal openings, as was coregistered during MRI and MEG) using a Polhemus device (FASTRAK, Polhemus Incorporated, Colchester, VT). For each subject, the locations of the digitized sensors were registered on the head model and MEG source estimate using landmark-based co-registration.

Using Helmholtz reciprocity, the individualized FEM forward models were also used for D-CMI mc-tDCS optimization as described by Wagner et al. [35] and Khan et al. [23] to maximize field intensity along the target-orientation, the so-called target directionality. This approach was chosen to enable stimulation with high target directionality while adhering to established safety constraints. The optimization ensured:

- High directionality at the target site.
- High parallelity of the electric field to the target dipole orientation.
- Reduction of side effects and skin-level sensations by current distribution over multiple stimulation electrodes.

Established safety constraints were imposed on the model: individual electrode current < 2 mA and total injected current ≤ 4 mA. Optimization was implemented using the CVX toolbox [36] (CVX, MATLAB) and following Khan et al. [23], we used regularization to add convexity to the optimization term and to distribute the currents across multiple electrodes. The output of the optimization algorithm for each individual included the recommended electrode montage, the number and positions of the cathodal and anodal electrodes on the EEG cap, and the stimulation intensity (mA) for each active electrode.

#### Application of mc-tDCS

To deliver stimulation, we used a Starstim-8 tDCS system (Neuroelectrics, Barcelona, Spain) with Ag/AgCl gel electrodes (Starstim NG Pistim). Each participant underwent three stimulation sessions in a randomized order. Before the first stimulation session, the investigator delivering mc-tDCS used MATLAB to generate the complete randomized sequence of the three conditions for each participant and knew the assigned condition before each session. Participants and the MEG technician conducting the recordings were not informed of the assignment. MEG with voluntary swallowing and a challenged swallowing task was performed before the interventions and after each intervention. Post-stimulation MEG recordings started 5 min after mc-tDCS to allow some time for repositioning in the MEG chamber. Stimulation conditions included: Low-Intensity anodal mc-tDCS (1.5 mA) and High-Intensity anodal mc-tDCS (4.0 mA) with the individually optimized electrode montages and stimulation parameters, and sham mc-tDCS with the same electrode montage as used in the low intensity condition. The current was gradually increased over 30 s, held at the target stimulation intensity for 9 min, and then gradually decreased. In the sham condition, the current was ramped up and down for 30 s to mimic a sensory effect (tingling) at the beginning of stimulation. Still, no current was sustained for the remaining 9 min [37]. We applied 1.5 mA based on prior evidence of physiological effects in swallowing-related networks, and 4.0 mA to explore intensity-dependent modulation within the device’s safety limits [9, 10, 29, 38]. During each mc-tDCS session, participants were instructed to suck on a lemon-flavored lollipop to activate sensory afferents and increase spontaneous swallowing rates. This concurrent sensory activation is intended to enhance the efficacy of tDCS by explicitly engaging the targeted swallowing network.

#### Integrity of the blind

After completion of each post-stimulation MEG recording, participants selected one of three response options – high-intensity, low-intensity, or sham – to indicate which stimulation condition they believed they had received; no unsure or don’t-know option was available. Blinding success was summarized using the percentage of correct identifications for each applied condition and agreement between perceived and applied conditions using Cohen’s κ.

### Behavioral data analysis

Behavioral data were analyzed using SPSS Statistics 28.0 (IBM Corp., USA). Descriptive statistics were used to characterize participants. Two-way repeated-measures ANOVAs with factors intervention type and time were used to ensure comparability of MEG task performance (head movement, swallow count) and to evaluate exploratory changes in submental EMG measures and timing accuracy (percentage of trials classified as ‘on time’) during the challenged task. The hit rate was considered a measure of performance on the experimental timing task, not a validated measure of swallowing function. Mauchly’s test was used to determine the sphericity assumption, and the Greenhouse–Geisser correction was applied in case of violation. If ANOVA identified significant main effects or interactions, post-hoc contrasts were constructed and compared across conditions using paired t-tests with Bonferroni correction for multiple comparisons. Statistical significance was set at *p* < 0.05. Effect sizes for the challenged-task hit-rate model were quantified as partial eta squared (ηp²) for Dose, Time, and Dose × Time.

## Results

### Feasibility, safety, and task performance

All participants completed all three stimulation sessions without dropouts. Stimulation was well tolerated, and no adverse events were reported during or after any of the mc-tDCS interventions. MEG task performance data are summarized in Table 1. Head movement and the number of analyzed swallowing events remained stable across baseline and post-intervention measurements, with no significant differences between conditions (all ANOVAs, *p* > 0.05), confirming comparable measurement conditions. For challenged-task hit rate, the repeated-measures analysis showed a significant main effect of Time (F(1,7) = 47.67, *p* < 0.001, ηp² = 0.872). The Dose main effect did not reach significance after Greenhouse–Geisser correction (F(2,14) = 3.57, *p* = 0.061, ηp² = 0.338), and the Dose × Time interaction was not significant (F(2,14) = 1.23, *p* = 0.319, ηp² = 0.150). These results support a general improvement across repeated task assessments but not a confirmed dose-specific behavioral effect. Submental EMG measures did not change during uncued swallowing. During challenged swallowing, a significant intervention type × time interaction was observed for EMG peak-to-peak amplitude. Post-hoc comparisons indicated increased peak-to-peak amplitude following low-intensity mc-tDCS compared to sham, which remained significant after Bonferroni correction (*p* = 0.036). RMS amplitude showed a similar pattern, reaching trend-level significance after correction. These behavioral and peripheral physiological measures primarily served as feasibility and control indices and were not considered as primary outcome measures of the study. No stimulation-related discomfort beyond transient tingling was reported.

**Table 1:** Swallow task performance & peripheral physiology in MEG

	Volitional swallow task		Challenged swallow task	
	Baseline	Post-intervention	Baseline	Post-intervention
*Head movement (cm)*				
mc-tDCS 1.5 mA	0.31 ± 0.19	0.36 ± 0.20	0.32 ± 0.29	0.28 ± 0.06
mc-tDCS 4 mA	0.31 ± 0.19	0.30 ± 0.09	0.29 ± 0.19	0.16 ± 0.06
sham	0.31 ± 0.12	0.29 ± 0.08	0.19 ± 0.08	0.18 ± 0.07
*Swallow count (n)*				
mc-tDCS 1.5 mA	70.0 ± 20.7	79.5 ± 25.2	39.3 ± 1.8	39.4 ± 1.8
mc-tDCS 4 mA	73.5 ± 23.4	76.6 ± 24.8	38.8 ± 2.8	39.4 ± 1.2
sham	73.0 ± 21.1	69.4 ± 30.5	39.1 ± 1.2	39.3 ± 1.2
*Percentage of hits during challenged swallow task (%)*				
mc-tDCS 1.5 mA			61.9 ± 8.2	76.4 ± 12.0
mc-tDCS 4 mA			63.6 ± 16.4	80.9 ± 11.7
sham			58.9 ± 16.9	66.4 ± 16.9
*Swallow duration based on EMG activity (s)*				
mc-tDCS 1.5 mA	1.5 ± 0.3	1.6 ± 0.4	1.1 ± 0.1	1.1 ± 0.1
mc-tDCS 4 mA	1.5 ± 0.3	1.4 ± 0.2	1.2 ± 0.3	1.2 ± 0.2
sham	1.5 ± 0.1	1.4 ± 0.2	1.1 ± 0.1	1.1 ± 0.1
*RMS amplitude (µV)*				
mc-tDCS 1.5 mA	44.3 ± 22.6	40.2 ± 12.2	46.8 ± 18.1	50.1 ± 19.8
mc-tDCS 4 mA	59.1 ± 51.7	42.2 ± 11.5	56.6 ± 26.9	47.3 ± 24.4
sham	43.2 ± 9.9	39.6 ± 9.8	53.2 ± 15.1	47.5 ± 11.4
*EMG peak-to-peak amplitude (µV)*				
mc-tDCS 1.5 mA	376.3 ± 162.5	372.3 ± 113.5	416.2 ± 173.0	446.0 ± 190.9
mc-tDCS 4 mA	479.2 ± 406.2	350.0 ± 86.3	424.1 ± 147.6	354.5 ± 147.6
sham	395.2 ± 72.1	369.7 ± 75.5	472.2 ± 129.6	427.3 ± 110.8

Data are presented in mean ± standard deviation. EMG: electromyography; MEG: magnetoencephalography; (mc-) tDCS: (multichannel-) transcranial direct current stimulation; RMS = root mean square

Blinding assessment revealed fair agreement between perceived and applied stimulation conditions (Cohen’s κ = 0.375, *p* = 0.009). Correct identification was highest for high-intensity mc-tDCS (75%); low-intensity mc-tDCS and sham were each correctly identified in 50% of sessions.

### MEG source reconstruction and targeting

**Fig. 2:**
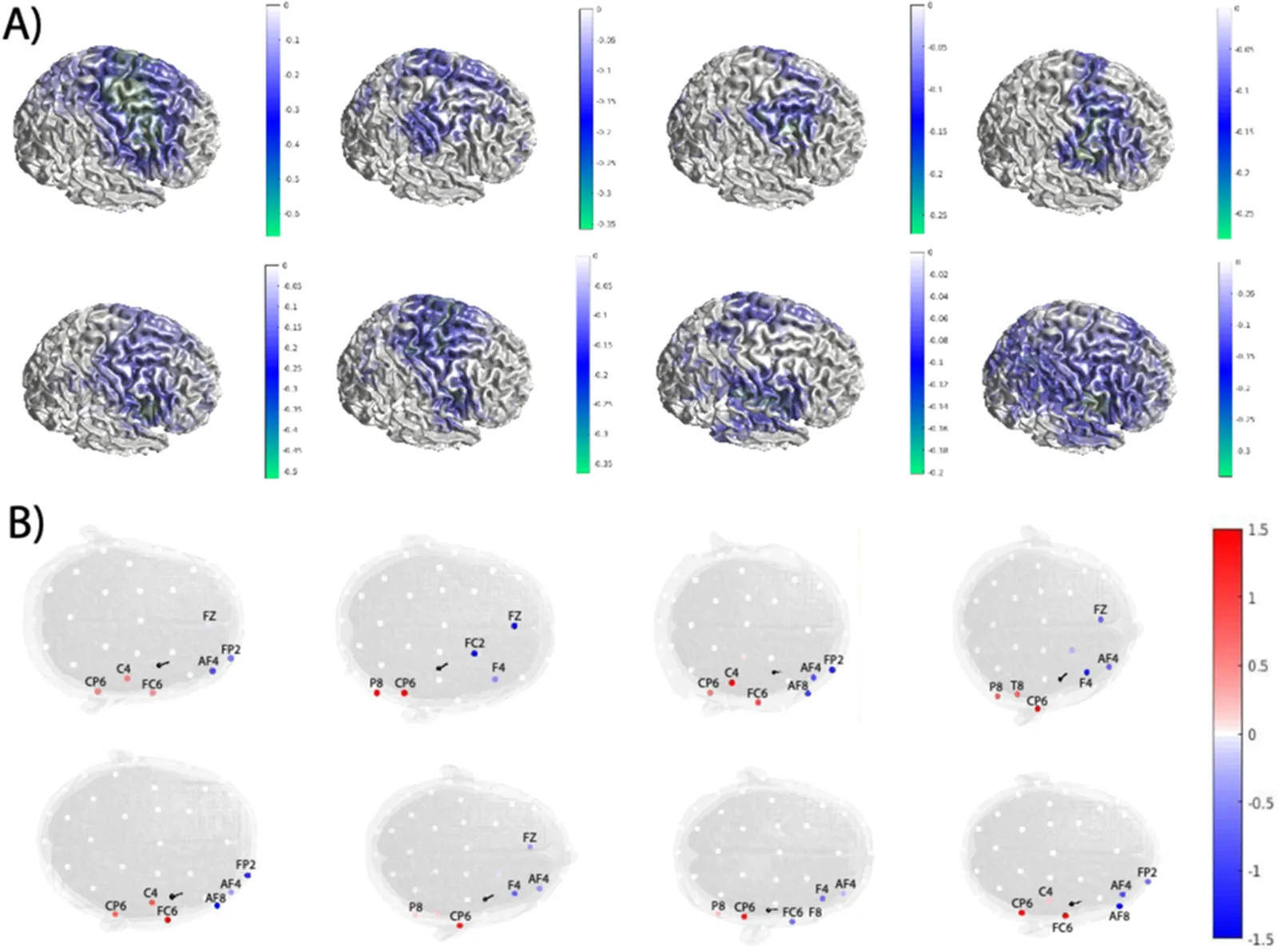
Individualized targeting and multichannel tDCS montage optimization. **(A)** Individual MEG source reconstructions illustrating swallowing-related cortical activity during uncued swallowing (β frequency band) in the right hemisphere for each participant. These activity patterns were used to identify the individual target region within the cortical swallowing network. **(B)** Individually optimized multichannel tDCS electrode montages derived using the distributed constrained maximum intensity (D-CMI) approach for each participant. Electrode positions are shown on the stimulation cap. Colors indicate electrode polarity (anodal: red; cathodal: blue), and color intensity reflects the applied current strength (mA) at each electrode. The visualization illustrates the inter-individual variability of optimized montages while maintaining consistent targeting of the swallowing-related cortical region

Uncued swallowing elicited the expected activation pattern of the cortical swallowing network, with prominent ERD in pericentral sensorimotor regions. This activity was most pronounced in the beta frequency range, which was therefore selected for individual target definition in the right hemisphere. Figure 2 illustrates the swallowing-related beta-band ERD for each participant, along with the corresponding target dipole derived from MEG source reconstruction and the individually D-CMI-optimized multichannel tDCS electrode montage.

### Baseline characterization of swallowing-related ERD

At baseline, swallowing elicited ERD across the theta to high-gamma frequency bands, with alpha- and beta-band oscillatory changes most pronounced across participants. Cortical activation was localized bilaterally in pericentral cortex, encompassing primary and secondary sensorimotor areas, with predominantly right-hemispheric involvement. In grand averages of baseline measurements, absolute peak ERD was most frequently observed in the beta band and was primarily localized in Brodmann areas 4 and 6 in the right hemisphere (Supplementary Table S2).

### Formal dose × task × time interaction analysis

No Dose × Task × Time interaction survived Holm correction across the five frequency bands: theta, F(2,14) = 0.51, Greenhouse–Geisser-adjusted *p* = 0.527, Holm-adjusted *p* = 1.000; alpha, F(2,14) = 0.72, *p* = 0.431, Holm-adjusted *p* = 1.000; beta, F(2,14) = 3.92, *p* = 0.055, Holm-adjusted *p* = 0.274; low gamma, F(2,14) = 0.56, *p* = 0.499, Holm-adjusted *p* = 1.000; and high gamma, F(2,14) = 0.21, *p* = 0.677, Holm-adjusted *p* = 1.000. The within-condition analyses below therefore characterize the observed responses but cannot by themselves establish differential effects between doses or tasks.

In an exploratory sensitivity analysis of the three band–task combinations with reported within-condition responses, the participant- and dose-adjusted period term was not significant for challenged alpha [F(2,12) = 0.51, Holm-adjusted *p* = 0.615], challenged beta [F(2,12) = 1.46, *p* = 0.545], or uncued low gamma [F(2,12) = 1.97, *p* = 0.545].

### Challenged swallowing: frequency-specific within-condition responses

In the separate within-condition analysis of the challenged swallowing task, low-intensity mc-tDCS was associated with a pre–post reduction in source-level ERD in the alpha band (*p* = 0.025) and beta band (*p* = 0.048). High-intensity mc-tDCS did not show significant pre–post changes in these bands (all *p* > 0.05). No significant pre–post ERD changes were observed in the gamma or theta bands for either active intensity or sham.

### Uncued swallowing: frequency-specific within-condition response

In the separate within-condition analysis of uncued swallowing, high-intensity mc-tDCS was associated with a pre–post change in low-gamma ERD (*p* < 0.001). Low-intensity mc-tDCS did not show significant pre–post ERD changes in any frequency band.

### Spatial distribution of within-condition ERD changes

**Fig. 3:**
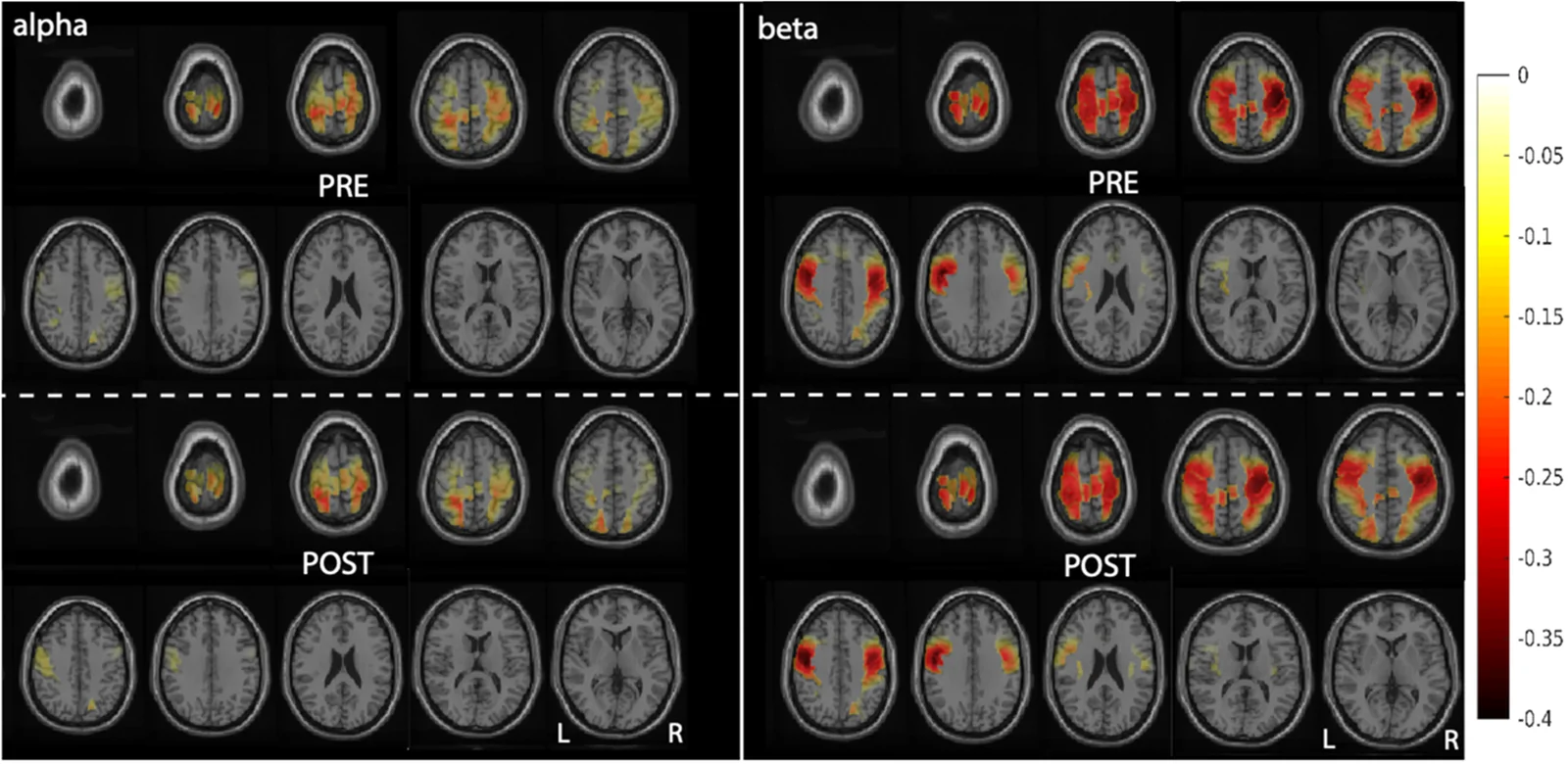
Grand average swallowing-related ERD maps during challenged swallowing (α and β bands). Grand average MEG-derived event-related desynchronization (ERD) maps are shown before (pre) and after (post) low-intensity mc-tDCS during the challenged swallowing task for the α (left) and β (right) frequency bands. The original 10-mm ROI analyses yielded significant clusters in both bands (α *p* = 0.025; β *p* = 0.048; cluster-based permutation testing within the predefined swallowing network). The maps demonstrate the spatial expression of these frequency-specific within-condition responses; the formal analysis did not confirm a difference between doses or tasks. Statistical cluster maps are shown in the Supplementary Material. Color coding indicates ERD magnitude. L = left hemisphere; R = right hemisphere

The spatial distribution of the pre–post changes was examined using cluster-based nonparametric statistics within the predefined swallowing-network ROI. During challenged swallowing, clusters associated with the low-intensity pre–post comparison were located primarily within bilateral sensorimotor and premotor cortical regions, including precentral and postcentral gyri and medial frontal areas; additional clusters were observed in parietal regions encompassed by the ROI. During uncued swallowing, clusters associated with the high-intensity pre–post comparison were located predominantly within sensorimotor cortical areas, with additional frontal and parietal involvement. Grand averages and cluster maps are presented in Fig. 3 and Supplementary Figures S2–S5. These maps show that the observed within-condition responses were spatially expressed within the predefined swallowing network.

## Discussion

This proof-of-concept study implemented a neuroengineering-guided approach integrating MEG-based target localization, subject-specific MRI-based finite-element modeling, and D-CMI-optimized multichannel stimulation. The hypothesized Dose × Task × Time interaction was not significant after Holm correction. Exploratory within-condition analyses identified alpha- and beta-band pre–post differences during challenged swallowing after 1.5-mA stimulation and a low-gamma difference during uncued swallowing after 4.0-mA stimulation. These observations describe changes within individual conditions; they do not establish stimulation-specific effects or differential responses across doses and tasks. The integrated workflow provides a methodological basis for further controlled investigation.

The separate within-condition patterns warrant cautious interpretation in relation to the processes engaged by each task. Alpha- and beta-band oscillatory activity within the swallowing network has previously been associated with sensorimotor integration, cortical resource allocation, and task-dependent control processes [10, 26, 31, 39–43]. Challenged swallowing additionally involved visual cue processing, precise timing, performance monitoring, and immediate feedback. These associations provide physiological context but do not establish which processes underlie the present exploratory pre–post differences. Although the cluster analyses controlled for multiple spatial comparisons within the predefined ROI [32], the separate within-condition comparisons do not establish stimulation-specific modulation or differential responses across doses and tasks. Because uncued swallowing always preceded challenged swallowing, task condition was also confounded with elapsed post-stimulation time and measurement order. Order and timing were identical across stimulation conditions, and pharyngeal corticobulbar after-effects have been reported for up to 60 min after a different tDCS protocol [9]; however, this does not resolve the task–time confounding in the present study. Whether individualized mc-tDCS interacts with ongoing network activity, as suggested by previous work in other settings [44], remains a question for future studies with counterbalanced task order and independently manipulated task components.

An important methodological aspect of the present study is the use of individualized, optimized multichannel stimulation based on a D-CMI approach [22, 23, 33]. Although swallowing is mediated by a distributed cortical network [45–47], targeting was deliberately based on a focal cortical site defined by its individual location and dominant dipole orientation derived from MEG source reconstruction. A key advantage of the D-CMI framework is its ability to enable high directional electric field strength along the target orientation while respecting safety and tolerability constraints [13, 22]. In contrast to maximum-intensity (MI) optimization, which can achieve maximal directionality at the cost of increased peripheral stimulation [48, 49], D-CMI incorporates regularization and side constraints that distribute current across multiple electrodes, thereby enabling near-maximal effective directionality with reduced skin sensations [22, 50]. The present study implemented this individualized targeting strategy for a focal cortical site within the distributed swallowing network. Future work should systematically compare optimization strategies along the intensity–focality continuum to delineate their physiological and practical trade-offs [51].

Previous studies indicate that the physiological effects of tDCS do not necessarily increase monotonically with applied current intensity [5, 6, 16–21, 52, 53]. Proposed mechanisms include homeostatic regulation, recruitment of inhibitory processes, and intensity-dependent changes in neuronal polarization; electric-field distribution, sensory effects, and expectancy may also influence responses. The present study did not establish differential effects across doses and tasks and therefore cannot determine whether these mechanisms contributed to the observed within-condition pre–post differences. The findings should consequently not be interpreted as evidence for a nonlinear dose–response or greater efficacy of 1.5-mA stimulation. Further dose-ranging studies incorporating measures of target engagement, sensation, and expectancy are needed to distinguish these possibilities.

The two task conditions provide a controlled experimental contrast but not a selective manipulation of a single network-state dimension. In addition, high-intensity stimulation was correctly identified in 75% of sessions. Cutaneous sensations, expectancy, attention, or arousal may therefore have influenced post-stimulation MEG activity. In particular, attentional interference during the more demanding challenged task or non-specific arousal during uncued swallowing are plausible alternative interpretations of the low-gamma pattern. However, the present data provide no direct evidence that these factors account for the observed cortical changes. Because trial-wise sensation and expectancy measures were not available for covariate analysis, non-specific and neuromodulatory contributions cannot be separated conclusively. Future studies should use improved active controls, collect graded sensation and expectancy ratings, and prespecify analyses that incorporate these variables.

Cortical pre–post changes were observed without a confirmed dose-specific change in behavioral task performance. Challenged-task hit rate improved over time across conditions, with a large Time effect (ηp² = 0.872), whereas the Dose effect and Dose × Time interaction were not significant. This pattern is compatible with repeated-task practice or familiarization and does not provide evidence of a stimulation-specific behavioral benefit. Whether the exploratory cortical pre–post differences have functional relevance remains unresolved. The challenged-task hit rate indexes performance on a laboratory timing task and is not a validated clinical measure of swallowing function. The submental EMG amplitude finding should be interpreted as exploratory. Future patient studies should combine neurophysiological target-engagement measures with clinically meaningful endpoints such as instrumental swallowing-safety and efficiency measures, validated dysphagia scales, nutritional or respiratory outcomes, and patient-reported function.

Although the present study was conducted in healthy participants and was not designed to assess clinical efficacy, the individualized workflow may have important implications for dysphagia rehabilitation. Post-stroke dysphagia is increasingly understood as a network disorder characterized by lesion-induced disruption and adaptive reorganization of cortical swallowing control [2, 29, 46, 54, 55]. Recovery depends on the recruitment and reweighting of residual network components, with substantial interindividual variability related to lesion location, structural connectivity, and compensatory mechanisms [54–60]. This variability may partly explain the heterogeneous results reported in clinical tDCS studies and meta-analyses [3, 11, 56, 57]. Individualized multichannel stimulation offers a potential strategy to address this variability by enabling targeted modulation of patient-specific network configurations. The present MEG/FEM/D-CMI workflow provides an experimental platform for examining how individualized targeting, electric-field orientation, stimulation intensity, and ongoing network activity jointly shape neurophysiological responses.

A potential translational pathway would involve several stages. Researchers could first examine target engagement and response reliability in a larger healthy cohort with stronger blinding and appropriate active comparators, including conventional bipolar or non-individualized multichannel stimulation. If target engagement proves reproducible, subsequent patient studies could assess feasibility, tolerability, and clinical effectiveness. Given the resource requirements of the full MEG/FEM/D-CMI pipeline, future work could additionally investigate whether targets derived from structural MRI combined with atlas-based priors or from task fMRI provide a sufficiently close approximation to MEG-informed targeting for broader clinical use. Prospective comparison of simplified and fully individualized workflows would be important before clinical implementation.

Several limitations should be considered. First, the sample size was small and limited to healthy young adults, restricting generalizability, precision, and power to detect interactions. Second, only acute after-effects of a single stimulation session were assessed, and it remains unclear whether repeated individualized stimulation induces cumulative or long-lasting plastic changes. Third, no conventional bipolar or non-individualized comparator was included, so effects cannot be attributed specifically to individualized targeting. Fourth, blinding was only partially preserved at 4.0 mA, and sensation, expectancy, attention, and arousal were not quantified for statistical adjustment [49]. Fifth, the task contrast combined cueing, timing, feedback, attentional, and motor differences and cannot isolate a single physiological state. The fixed task order confounded task condition with elapsed post-stimulation time and measurement order. No evidence of a period effect was detected in the targeted sensitivity analysis; given the sample size, residual period or carryover effects cannot be ruled out. Sixth, behavioral measures were exploratory and did not include validated clinical swallowing outcomes. Finally, the resource-intensive MEG/FEM/D-CMI pipeline and focus on a single cortical target limit immediate clinical scalability. These constraints reinforce the hypothesis-generating nature of the descriptive pre–post findings.

In conclusion, this study demonstrates the feasibility of integrating functional neuroimaging, computational head modeling, and optimized electric-field targeting and reports exploratory frequency-specific pre–post differences within the cortical swallowing network. The formal interaction analysis did not confirm differential effects across doses and tasks; the within-condition findings therefore remain hypothesis-generating and do not establish stimulation-specific modulation. These findings support targeted replication in larger cohorts with strengthened blinding, active comparator conditions, behavioral effect estimates, and clinically meaningful outcomes.

## Supplementary Information

Below is the link to the electronic supplementary material.

Supplementary Material 1

Supplementary Material 2

Supplementary Material 3

Supplementary Material 4

Supplementary Material 5

Supplementary Material 6

Supplementary Material 7

## Data Availability

The datasets and analysis code supporting the conclusions of this article are available from the corresponding author upon reasonable request. Due to institutional and data protection regulations of the University Hospital Münster, data sharing requires a formal data-sharing agreement.

## Abbreviations

ACC: Anterior cingulate cortex
BA: Brodmann area
D–CMI: Distributed constrained maximum intensity
EMG: Electromyography
ERD: Event–related desynchronization
FEM: Finite element method
LCMV: Linearly constrained minimum variance
MEG: Magnetoencephalography
MRI: Magnetic resonance imaging
mc–tDCS: multichannel transcranial direct current stimulation
ROI: Region of interest
tDCS: transcranial direct current stimulation
