# Supplementary material for: Frequency-specific cortical swallowing-network responses following neuroengineering-guided individualized multichannel tDCS
Source: J Neuroeng Rehabil. 2026 Nov 12;23:320. doi: 10.1186/s12984-026-02214-0 (PMC13761166; doi:10.1186/s12984-026-02214-0)
Supplement: Supplementary file 1 [file 12984_2026_2214_MOESM1_ESM.pdf]

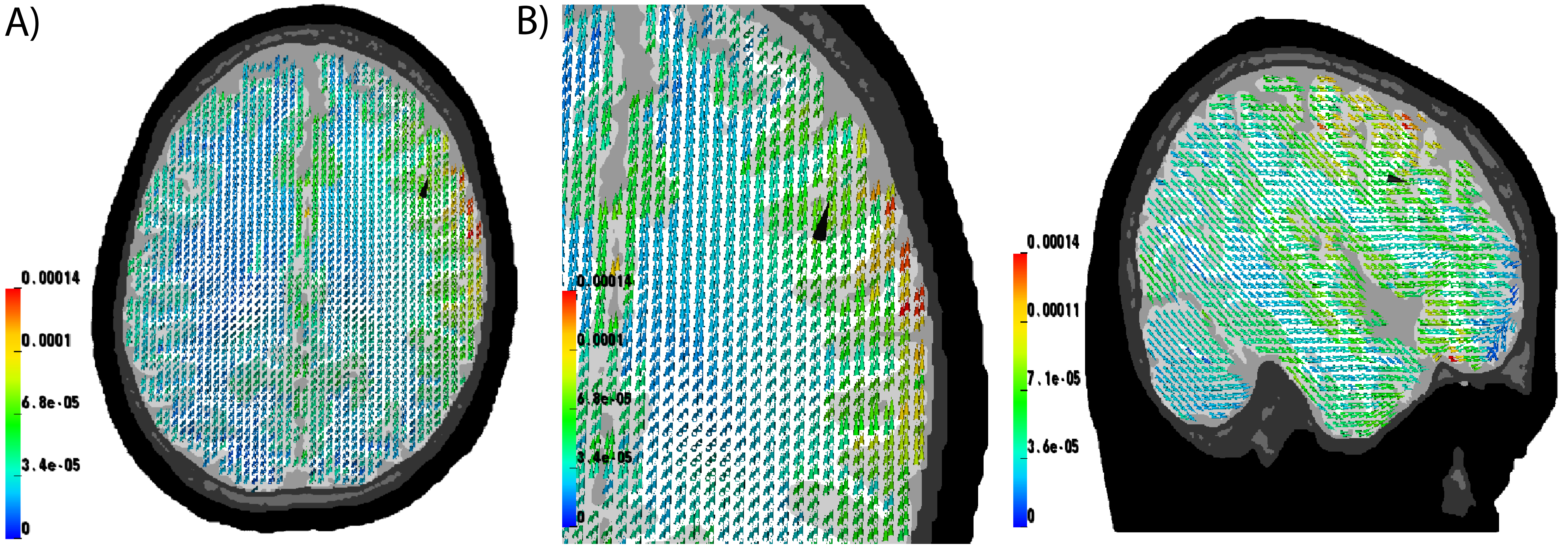

A)
B)
0.00014
0.0001
6.8e-05
3.4e-05
0
0.00014
0.0001
6.8e-05
3.4e-05
0
0.00014
0.00011
7.1e-05
3.6e-05
0
