# Supplementary material for: Frequency-specific cortical swallowing-network responses following neuroengineering-guided individualized multichannel tDCS
Source: J Neuroeng Rehabil. 2026 Nov 12;23:320. doi: 10.1186/s12984-026-02214-0 (PMC13761166; doi:10.1186/s12984-026-02214-0)
Supplement: Supplementary file 2 [file 12984_2026_2214_MOESM2_ESM.pdf]

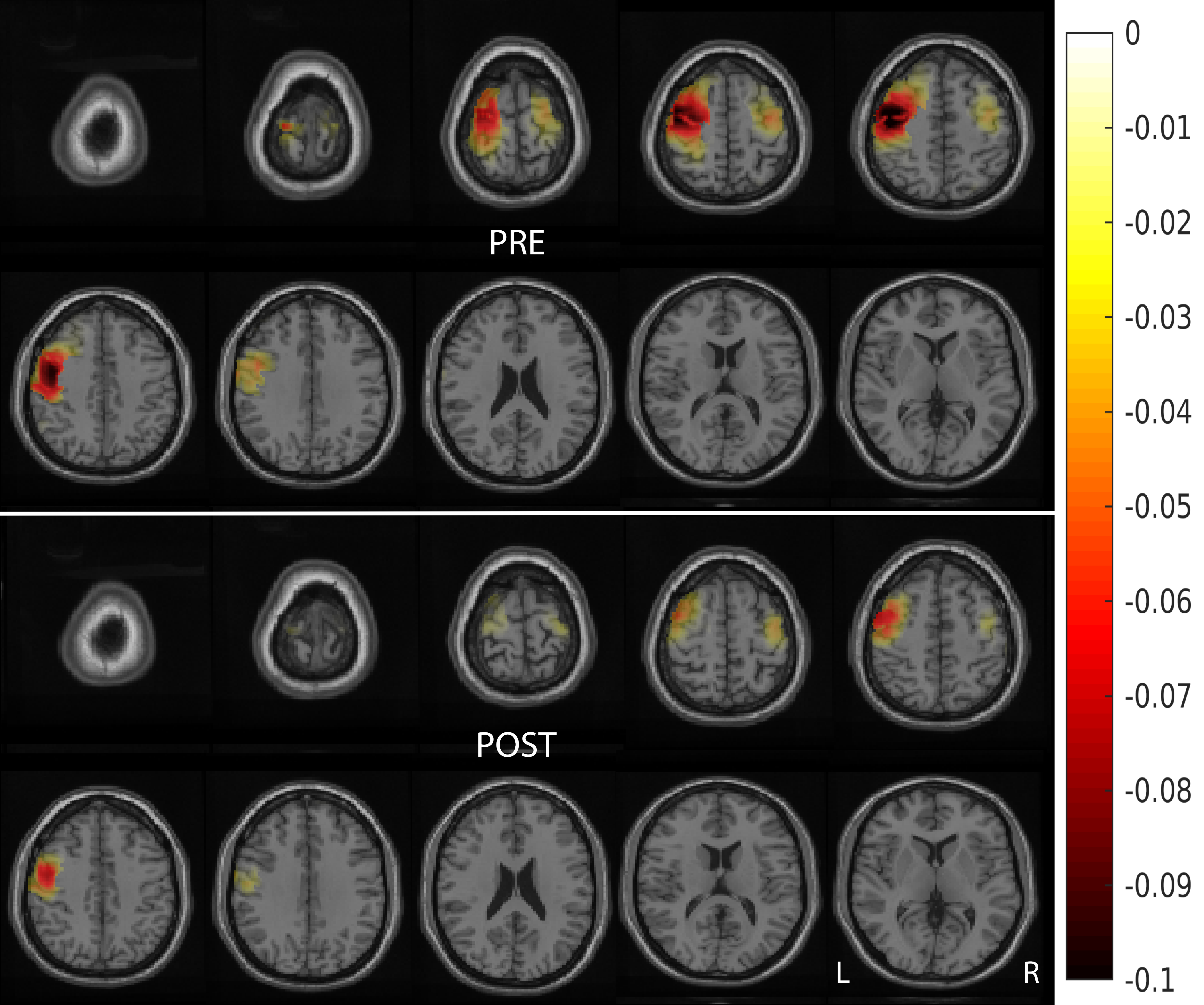
PRE
POST
L
R
0
-0.01
-0.02
-0.03
-0.04
-0.05
-0.06
-0.07
-0.08
-0.09
-0.1
