# Supplementary material for: Frequency-specific cortical swallowing-network responses following neuroengineering-guided individualized multichannel tDCS
Source: J Neuroeng Rehabil. 2026 Nov 12;23:320. doi: 10.1186/s12984-026-02214-0 (PMC13761166; doi:10.1186/s12984-026-02214-0)
Supplement: Supplementary file 3 [file 12984_2026_2214_MOESM3_ESM.pdf]

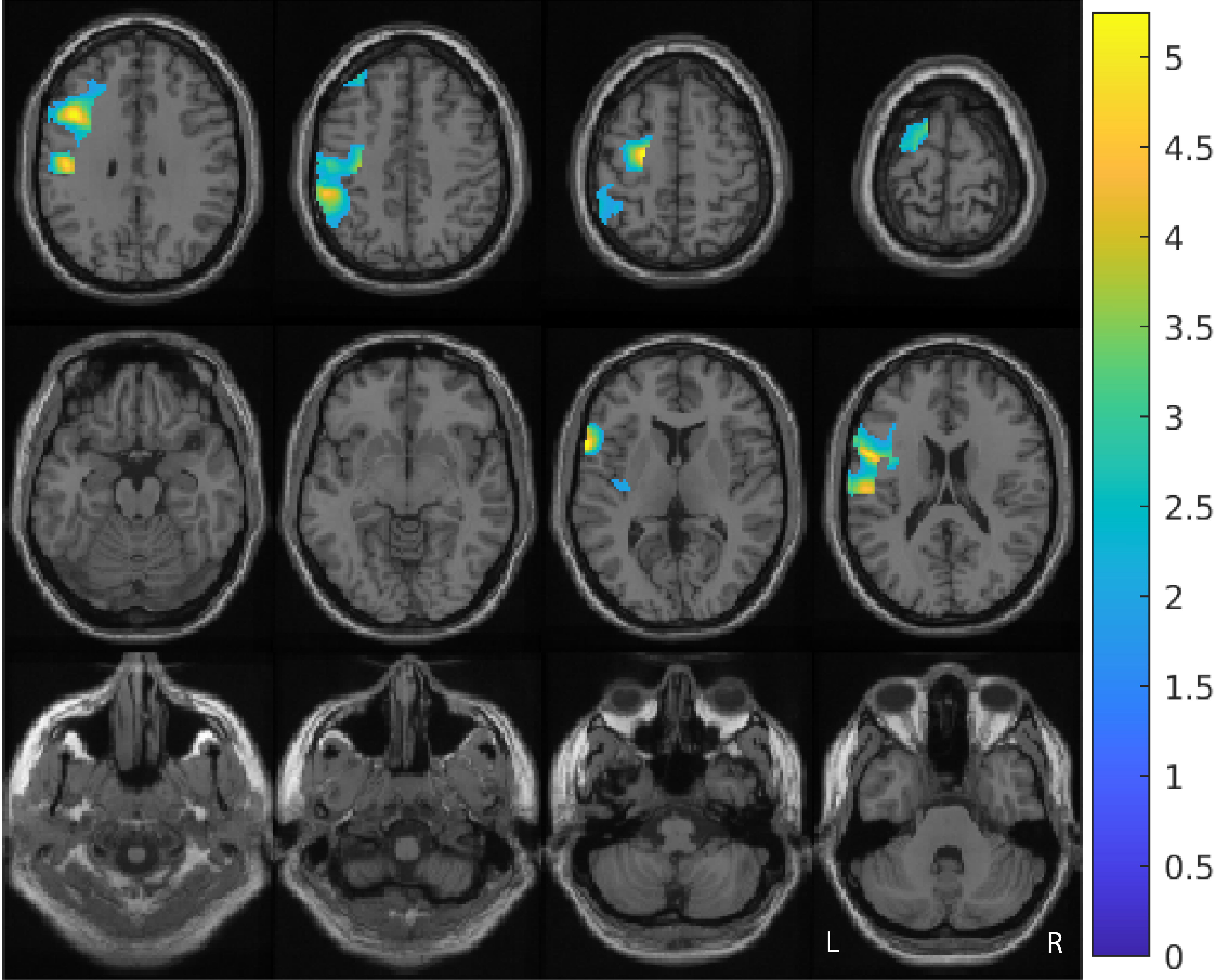
L
R
0
0.5
1
1.5
2
2.5
3
3.5
4
4.5
5

L
