# Supplementary material for: Frequency-specific cortical swallowing-network responses following neuroengineering-guided individualized multichannel tDCS
Source: J Neuroeng Rehabil. 2026 Nov 12;23:320. doi: 10.1186/s12984-026-02214-0 (PMC13761166; doi:10.1186/s12984-026-02214-0)
Supplement: Supplementary file 6 [file 12984_2026_2214_MOESM6_ESM.docx]

**Supplementary Figures – Legends**

**Supplementary Figure S1.** **Exemplary vector field representations of the simulated electric field resulting from D-CMI–optimized multichannel tDCS.**

Different cross-sectional views illustrate the orientation and spatial distribution of the electric field relative to the individualized target.

**Supplementary Figure S2.** **Grand average swallowing-related cortical activity during uncued swallowing (low-γ band)**

Grand average MEG-derived event-related desynchronization (ERD) maps are shown before (pre) and after (post) high-intensity mc-tDCS during uncued swallowing in the low-γ frequency band. Maps depict group-level average cortical activity without statistical masking. Group-level statistical analysis revealed significant pre–post differences for this condition(p <0.001) (see Supplementary Figure S5 for corresponding statistical maps). Color coding indicates ERD magnitude. L = left hemisphere; R = right hemisphere

**Supplementary Figure S3. Statistical cluster map of pre–post modulation during challenged swallowing (α band)**

Statistical maps illustrate clusters showing significant pre–post differences in MEG-derived ERD following low-intensity mc-tDCS during the challenged swallowing task in the α frequency band. Results are based on cluster-based permutation testing within the predefined swallowing network (p = 0.025). Color coding reflects t-values. Only clusters reaching statistical significance are shown.

**Supplementary Figure S4. Statistical cluster map of pre–post modulation during challenged swallowing (β band)**

Clusters showing significant pre–post differences in ERD following low-intensity mc-tDCS during challenged swallowing in the β frequency band, identified using cluster-based permutation testing within the swallowing network (p = 0.048). Color coding indicates t-values. Only clusters reaching statistical significance are shown.

**Supplementary Figure S5. Statistical cluster map of pre–post modulation during uncued swallowing (low-γ band)**

Statistical clusters indicate significant pre–post changes in ERD following high-intensity mc-tDCS during uncued swallowing in the low-γ frequency band (cluster-based permutation testing, p < 0.001). Only statistically significant clusters are displayed.
