# Supplementary material for: Frequency-specific cortical swallowing-network responses following neuroengineering-guided individualized multichannel tDCS
Source: J Neuroeng Rehabil. 2026 Nov 12;23:320. doi: 10.1186/s12984-026-02214-0 (PMC13761166; doi:10.1186/s12984-026-02214-0)
Supplement: Supplementary file 7 [file 12984_2026_2214_MOESM7_ESM.docx]

***Supplementary Tables***

**Supplementary Table S 1 Predefined cortical regions of interest included in the swallowing network analysis**. Regions were selected a priori based on previous neuroimaging studies of swallowing-related cortical activity.

| **Cortical region** | **Abbreviation** |
| --- | --- |
| Inferior frontal gyrus | IFG |
| Medial frontal gyrus | MFGmed |
| Middle frontal gyrus | MFG |
| Superior frontal gyrus | SFG |
| Precentral gyrus | PreCG |
| Postcentral gyrus | PostCG |
| Paracentral lobule | PCL |
| Anterior cingulate cortex | ACC |
| Insula | INS |
| Superior parietal lobule | SPL |
| Inferior parietal lobule | IPL |

***Supplementary Table S 2 Descriptive anatomical localization of peak swallowing-related cortical activity.*** The table summarizes the approximate Brodmann area and anatomical region corresponding to the peak cortical activity during swallowing in the α and β frequency bands for the uncued and challenged swallowing conditions. Peak locations were derived from grand average MEG source reconstructions and are provided for descriptive purposes only.

| **Conditions** | | **Uncued swallowing** | | **Challenged swallowing** | |
| --- | --- | --- | --- | --- | --- |
|  |  | alpha | beta | alpha | beta |
| **sham** | pre | Right BA 6: -0.118 | Right BA 6: -0.222 | Right BA 1: -0.299 | Right BA 4: -0.389 |
|  | post | Right BA 6: -0.164 | Right BA 6: -0.255 | Right BA 1: -0.229 | Right BA 6: -0.413 |
| **1.5 mA** | pre | Right BA 6: -0.139 | Left BA 4: -0.218 | Right BA 5: -0.259 | Left BA 4: -0.394 |
|  | post | Left BA 4: -0.154 | Right BA 6: -0.271 | Right BA 5: -0.291 | Right BA 6: -0.399 |
| **4.0 mA** | pre | Right BA 6: -0.094 | Right BA 6: -0.234 | Right BA 1: -0.270 | Left BA 6: -0.370 |
|  | post | Right BA 6: -0.089 | Right BA 6: -0.200 | Right BA 6: -0.298 | Right BA 6: -0.353 |
